# Knowledge, Attitudes, and Practices Regarding Cervical Cancer Screening among HIV-infected Women at Srinagarind Hospital: A Cross-Sectional Study

**DOI:** 10.31557/APJCP.2020.21.10.2979

**Published:** 2020-10

**Authors:** Athiwat Songsiriphan, Lingling Salang, Woraluk Somboonporn, Nuntasiri Eamudomkarn, Wilasinee Nhokaew, Chusri Kuchaisit, Pornnipa Harnlakorn

**Affiliations:** 1 *Department of Obstetrics and Gynecology, Faculty of Medicine, Khon Kaen University, Khon Kaen, Thailand. *; 2 *Retired Government Official, Nursing Division, Srinagarind Hospital, Faculty of Medicine, Khon Kaen University, Khon Kaen, Thailand. *; 3 *Senior Professional level AIDs Unit, Nursing Division, Srinagarind Hospital, Faculty of Medicine, Khon Kaen University, Khon Kaen, Thailand. *

**Keywords:** Knowledge, attitudes, practices, cervical cancer screening, HIV, infected women

## Abstract

**Introduction:**

In recent years, the lives of HIV-infected patients in Thailand have improved significantly due to continuous advances in treatment. However, the rate of cancer related to HIV infection (especially cervical cancer) is likely to increase. Although the World Health Organization (WHO) recommends Papanicolaou testing in all HIV-infected women, few of these patients receive this kind of screening in Thailand. Therefore, we conducted this study to evaluate the knowledge, attitudes, and practices of these patients with regard to cervical cancer screening.

**Materials and Methods:**

This cross-sectional study was conducted in HIV-infected women aged 18-65 years from April to November 2019 via a self-administered cervical cancer screening questionnaire, which consisted of four parts: demographic data, knowledge, attitudes, and practices.

**Results:**

Three hundred HIV-infected women were recruited. Most of the participants had good attitudes toward screening and practiced adequate screening (75.3% and 71.3%, respectively). However, only 62 participants (20.7%) demonstrated adequate knowledge. The crucial factors that were associated with adequate screening practice were age 40-49 years-old (AOR =3.26, 95%CI=1.02-10.37), CD4 cell count (AOR = 3.41, 95%CI = 1.29-8.99), having been advised about cervical cancer screening (AOR= 6.23, 95%CI 1.84-21.07), and attitude toward screening (AOR= 5.7, 95%CI = 2.23-14.55). The major reasons for not undergoing screening were embarrassment (41.86%), lack of symptoms (41.86%), fear of the results (36.04%), and fear of pain (36.04%).

**Conclusion:**

The reasons for inadequate testing were disregard and misconceptions about the procedure. To prevent invasive cervical lesions in HIV-infected women, health care providers should inform these patients about the importance of regular cervical cancer screening.

## Introduction

Human immunodeficiency virus (HIV) is a transmittable disease that affects the immunity of its host. Currently, there are many anti-retroviral drugs available to decrease viral load including highly active antiretroviral therapy (HARRT). However, while this regimen is effective in decreasing viral load, it does not reduce the risk of other long-term complications such as malignancy-related HIV infection (Kietpeerakool, 2005). Women who have coinfection with HIV and human papillomavirus (HPV) have a greater chance of cervical transformation. Previous studies have found that HIV-infected women had a 6.7 times greater risk of abnormal cytological test results and four times greater risk of cervical cancer than those without HIV infection (Vafaei et al., 2015; Abraham et al., 2013). 

In Thailand, cervical cancer was the HIV-related malignancy with the highest incidence (Kiertiburanakul et al., 2007). A previous study found the prevalence rates of HPV infection, abnormal cytological test results, and cervical cancer in these patients to be 38.6%, 20.4%, and 1.9%, respectively (Sirivongrangson et al., 2007), suggesting that HIV-infected women had an increased risk of cervical cancer. Despite this, another study found that only 14.7% of HIV-infected women undergo Pap smear screening (Chen et al., 2013).

According to GLOBOCAN (2018), cervical cancer was the third most common cancer among women worldwide. In 2018, there were 569,000 new cases of cervical cancer, or 13.1 patients per 100,000, with 311,400 deaths (7.5% of all female cancer deaths; Bray et al., 2018). Previous studies conducted in Thailand found that cervical cancer was the second most common malignancy in women, with 8,622 new cases and an incidence of 16.2 patients per 100,000 in 2018. In total, 5,015 patients die of cervical cancer annually (an average of 13 patients per day; Chaowawanit et al., 2016.; Bray et al., 2018) 

Human papillomavirus is a sexually transmitted disease, some serotypes of which (especially HPV 16 and HPV 18) are major causes of cervical cancer. Although the immune response in most HPV-infected patients leads to remission, some patients experience persistent infection and develop cervical cancer within 10-20 years (Saslow et al., 2012; Walboomers et al., 1999). Because it has a long preinvasive period, invasive cervical cancer is considered a preventable disease. If a preinvasive lesion is detected and treated early, the risks of morbidity and mortality decrease significantly (Rukrungtam et al., 2017; Anderson et al, 2019). 

The aims of this study were to evaluate the knowledge, attitudes, and practices of HIV-infected women in Thailand with regard to cervical cancer screening and to evaluate the factors that affect whether or not these patients undergo screening. 

## Materials and Methods

This cross-sectional study was conducted in HIV-infected women at Srinagarind Hospital in Khon Kaen, Thailand. Prior to the study, a questionnaire was developed to evaluate knowledge, attitudes, and practices with regard to cervical cancer screening, which was then tested in 20 HIV-infected women who sought medical care at Srinagarind Hospital’s Infectious Disease Clinic as a pilot study. The final questionnaire was validated by three gynecologic oncologists (not involved in the study). The study, conducted from April to November 2019, was approved by the Human Research Ethics Committee of Khon Kaen University as per the Helsinki Declaration and Good Clinical Practice Guidelines (HE621021). 

The primary endpoint of this study was to evaluate knowledge, attitudes, and practices of HIV-infected women in Thailand with regard to cervical cancer screening. A previous study by Chaowawanit et al., (2016) found that 26.4% of women had good attitudes about screening and adequate knowledge. Another study by Budkaew et al., (2014) found that 32.3% of women underwent cervical screening. Assuming a non-response rate of 5%, we estimated that a sample size of 300 participants would be required.

 Women who had been diagnosed with HIV at least five years prior, were aged 18 to 65, underwent follow-up examination at Srinagarind Hospital’s Infectious Disease Clinic, and could read and write in Thai were included in this study. We excluded women with history of pre-invasive or invasive cervical lesions or other gynecologic cancers, hysterectomy for any reason, or HPV vaccination, or who were pregnant at the time of the study, or who could not provide the necessary information.

An information sheet about the project was given to all participants who met the inclusion criteria. A verbal explanation was also provided if anything was unclear. All women gave verbal consent and completed a Thai-language questionnaire, either by themselves with assistance if necessary for comprehension. Any questions about the questionnaire were answered by our research assistants before proceeding. 

*Questionnaire design*

The questionnaire was a revised version of that used in a study by Chaowawanit et al. (2016). It was validated by three experts (not involved in this study), and its reliability was tested in a pilot study with 20 patients. Its reliability for knowledge outcomes as calculated using the Kuder-Richarson coefficient (KR20) was 0.77 and for attitude outcomes according to Cronbach’s alpha was 0.84 (similar to the 0.78 found in a previous study by Charoenmak et al. (2013)). A KR-20 coefficient and Cronbach’s alpha equal to or greater than 0.7 and 0.75, respectively, were considered acceptable (Anderson et al., 2002; Feldt, 1965).

*Data collection*

The questionnaire was divided into four parts: 1) demographic data, 2) knowledge regarding cervical cancer screening, 3) attitudes about cervical cancer screening, and 4) screening practice. It required 30 minutes to complete and was self-administered in a private room. Research assistants collected the finished questionnaires and checked them for completion. 

There were 13 questions in the knowledge section, each worth 1 point. We used modified Bloom’s cut-off points (Narayana et al., 2017) to classify knowledge level, with scores of 0-9 indicating “inadequate knowledge” and 10-13 indicating “adequate knowledge.” The attitude section consisted of 10 questions, which were adapted from the Health Belief Model, each worth up to 4 points. A score of 10-30 was classified as a “poor attitude” and 31-40 as a “good attitude.” Screening practice was classified into three categories according to Thailand’s 2017 national guidelines on HIV/AIDS treatment and prevention: 1) adequate 2) inadequate, and 3) never. Adequate practice was defined as annual screening if CD4 levels are <500 cell/mm^3^ and cytological testing every two years and co-testing every 3 years if CD4 levels were ≥500 cell/mm3. Inadequate practice was defined as having undergone screening but less frequently than was considered adequate. 

*Statistical analysis*

We used STATA/SE version 10.1 to analyze participants’ baseline characteristics, knowledge, attitudes, and practice in terms of mean, median, percentage, standard deviation (SD), and 95% confidence interval (95% CI). Univariate analysis using a chi-square test, Fisher’s exact, and t-test was conducted to evaluate the associations between socio-demographic characteristics and knowledge, attitudes, and practice. We performed binary logistic regression analysis to predict knowledge, attitudes, and practice using binary and multinomial logistic regression. A p-value < 0.05 and odds ratio with a 95% CI were used to determine any associations.

## Results

Three-hundred HIV-infected women were recruited for this study. The baseline characteristics were presented in Table 1. The mean age of the participants was 45 ± 9.5 years, with most (68%) under 50 years of age. Most were married, and the average age at first intercourse was 22.2 years. The most common occupations were farmer, company employee, and public servant (35%, 29.6%, and 22.6% of participants, respectively). In terms of education, 46.3% held a bachelor’s degree or higher. Most were non-smokers (97%), Buddhist (98%), multiparous (44.9%), and had menstrual period (65.7%). Most (82.7%) used contraception such as oral contraceptive pills (OCPs) (47.3%), condoms (44.3%), and tubal resection (TR; 22.3%). The average duration since HIV diagnosis was 12.8±7.4 years. The mean CD4 level was 533.8±285.5 cell/mm^3^, and 148 (49.3%) participants had CD4 levels of less than 500 cell/mm^3^. The route of HIV infection in the majority of cases (90.3%) was sexual intercourse. Two hundred seventy-six of the participants (92%) had been advised about cervical cancer screening. 

*Overall Evaluation of Knowledge, Attitude, and Practice*

The average knowledge and attitude scores were 6.39 ±3.18 points and 33.98 ±5.39, respectively. Although only 62 participants (20.7%) demonstrated adequate knowledge of cervical cancer screening, 226 (75.3%) had good attitude scores. Cervical cancer screening practice was adequate in 214 participants (71.3%), inadequate in 55 participants (18%), and 31 (10.3%) had never undergone screening (Table 2).

*Factors associated with Attitude and Knowledge*

High level of education, more pregnancies, contraception use, and high income were significantly correlated with adequate knowledge. However, only education level and number of pregnancies remained after multivariable regression analysis. Women with a bachelor’s degree or higher were 2.24 times more likely to have adequate knowledge than those without (adjusted odds ratio [AOR]= 2.41; 95%CI = 1.4-4.4). A higher number of parities was also significantly associated with adequate knowledge (parity = 1; AOR = 4.1, 95%CI = 1.5 - 11.23 and parity ≥ 2; AOR = 3.67, 95%CI =1.37-9.83). However, high income and contraceptive use were less likely to be associated (AOR= 1.67, 95%CI = 0.74-3.76; AOR = 2.24, 95%CI = 0.82-6.981, respectively; Table 3). 

Number of parities and history of sexual intercourse were significantly associated with attitude toward cervical cancer screening according to multivariable regression analysis. Women who had been pregnant at least once were about 2-3 times more likely to have a good attitude than those who had not (parity = 1: AOR = 3.39, 95%CI = 1.55-7.42; parity ≥ 2: AOR = 2.11, 95%CI = 1.07-4.19), and those with a history of sexual intercourse were 3.36 times more likely than those who had never had sexual intercourse (AOR = 3.36, 95%CI=1.07-10.67). 

*Factors associated with Cervical Cancer Screening Practice*

The associated factors related to cervical cancer screening practice were age, parity, mean age at first intercourse, CD4 level, whether or not the patient had been advised regarding cervical cancer screening, and attitude toward screening. 

Participants aged 40-49 years were 3.26 times more likely to practice adequate screening compared to women < 40 years old (AOR = 3.26, 95%CI = 1.02-10.37), while women ≥ 50 years of age were 2.35 times more likely than those under 40 (AOR = 2.35, 95%CI = 0.67-8.32). However, there were no significant differences in the proportions of those practicing inadequate screening versus never undergoing screening by age group.

Although there was no statistically significant association between parity and cervical cancer screening practice, those who had been pregnant once or were multiparous more likely to practice adequate screening than those who were nulliparous (AOR = 3.33, 95%CI = 0.96-11.49 and AOR =2.02, 95%CI = 0.71-5.78, respectively). They were also more likely to practice inadequate screening than to not undergo screening.

There was a significant difference in terms of mean age at first intercourse between women who practiced adequate screening and those who had never been screened. Those who first engaged in intercourse at ≥ 20 years old were 1.10 times more likely to practice adequate versus inadequate screening than those who did so when they were < 20 (AOR= 1.10, 95%CI =1.01-1.19). However, there was no statistically significant correlation between age at first intercourse and inadequate versus no screening (AOR=0.89, 95%CI =0.31-2.52). 

Participants with CD4 levels greater than 500 cell/mm^3^ were 3.41 times more likely to practice adequate versus no screening than women with CD4 levels < 500 cell/mm^3^ (AOR = 3.41, 95%CI=1.29-8.99), but there were slightly significant difference when comparing between inadequate practice and never practice (AOR = 0.61, 95%CI=0.21-1.79) . Not having been advised about cervical cancer screening was a major factor associated with non-adherence in terms of both adequate screening versus never having been screened (AOR = 6.32, 95%CI = 1.84-21.07) and inadequate screening versus never having been screened (AOR = 2.62, 95%CI = 0.75-9.19). 

Women with good attitudes toward screening were 5.7 times more likely to practice adequate versus no screening compared with those who had poor attitudes (AOR = 5.7, 95% CI = 2.23 – 14.55). Conversely, those with good attitudes were less likely to practice inadequate versus no screening than those with poor attitudes (AOR = 1.35, 95%CI=0.51-3.59).

By contrast, women with a history of pregnancy were less likely than nulliparous women to practice adequate versus no screening (AOR = 3.33, 95%CI = 0.96-11.49). This was also true for multiparous versus nulliparous women (AOR=2.20, 95%CI = 0.71-5.78) in comparing in both adequate versus no screening and inadequate versus no screening, as shown in Table 4.

*Reasons for inadequate cervical cancer screening*

The 86 participants who practiced inadequate screening gave the following reasons: embarrassment, lack of symptoms, fear of pain, fear of the results, feeling they were not at risk, cost, feeling that screening was unnecessary, and having a bad impression of health services (41.86%, 41.86%, 36.04%, 36.04%, 29.07%, 27.91%, 25.58%, and 12.79%, respectively; Table 5).

**Table 1:** Sociodemographic Data

Characteristics	Data
Mean age, year (2SD)	45 (± 9.5)
Marital status	
Single, n (%)	76 (26.3)
Married, n (%)	162 (54)
Divorced, n (%)	59 (19.7)
Occupation	
Public servant, n (%)	78 (22.6)
Employee, n (%)	89 (29.6)
Unemployed/Housewife, n (%)	27 (9)
Farmer, n (%)	106 (35)
Education level	
Primary school, n (%)	49 (16.3)
High school, n (%)	110 (36.6)
Bachelor’s degree, n (%)	120 (40)
Master’s degree or higher, n (%)	19 (6.3)
Age	
<50 years n (%)	204 (68)
≥50 years n (%)	96 (32)
Smoking status	
Yes n (%)	9 (3)
No n (%)	291 (97)
Religion	
Buddhist, n (%)	294 (98)
Christian, n (%)	4 (1.3)
Others, n (%)	2 (0.67)
Income (Baht)/month	
<10,000, n (%)	87 (29)
10,000-20,000, n (%)	96 (32)
20,001-30,000, n (%)	39 (13)
≥30,001, n (%)	78 (26)
Parity	
0, n (%)	72 (24)
1, n (%)	93 (31)
2, n (%)	97 (32.3)
3, n (%)	31 (10.3)
≥4, n (%)	7 (2.3)
Menstruation	
Yes, n (%)	197 (65.7)
No, n (%)	103 (34.3)
Sexual intercourse	
Yes, n (%)	286 (95.3)
Average age at first intercourse	22.2 (years)
Median number of partners	2 partners
Contraception history	
Yes, n (%)	248 (82.7)
Condom, n (%)	133 (44.3)
Oral contraceptive pills, n (%)	142 (47.3)
Tubal ligation, n (%)	67 (22.3)
Characteristics	Data
HIV infection history	
Duration of diagnosis, Mean ± SD	12.8±7.4
CD4 level, Mean ± SD	533.8±285.5
CD<500 cell/mm3, n (%)	148 (49.3)
CD≥500 cell/mm3, n (%)	152 (50.7)
Route of infection	
Sexual intercourse, n (%)	271 (90.3)
Blood, n (%)	20 (6.7)
Others, n (%)	9 (3)
Advised about cervical cancer screening	
Yes, n (%)	276 (92)
No, n (%)	24 (8)

**Table 2:** Knowledge, Attitudes, and Practice Regarding Cervical Cancer Screening in HIV-Infected Women

characteristics	N (%)	95% confident interval
Knowledge		
Knowledge (MD±SD)	6.39 ±3.18	6.02-6.75
Poor [0-9], n (%)	238 (79.3)	74.3-83.77
Good [10-13], n (%)	62 (20.7)	16.23-25.70
Attitude		
Attitude (MD±SD)	33.98 ±5.39	33.37-34.60
Poor [10-30], n (%)	74 (24.7)	21.49-29.95
Good [31-40], n (%)	226 (75.3)	70.05-80.11
Practice		
Adequate, n (%±SD)	214 (71.3±2.6)	66.19-76.48
Inadequate, n (%±SD)	55 (18±2.2)	13.93-22.73
Never, n (%±SD)	31 (10.3±1.8)	6.87-13.80

**Table 3:** Association with Knowledge and Attitude Regarding Cervical Cancer Screening

Variable	Outcome		Crude Odds ratio (95%CI)	Adjusted Odds ratio (95%CI)
	Poor n (%)	Good N (%)		
Knowledge of cervical cancer screening				
Education				
< Bachelor’s degree	31 (16.40)	158 (83.60)	1	1
≥ Bachelor’s degree	31 (27.93)	80 (72.7)	1.96 (1.12 - 3.48)	12.24 (1.4 - 4.4)
Number of parities				
0	6 (8.3)	66 (91.7)	1	1
1	24 (25.8)	69 (74.2)	3.83 (1.47 - 9.95)	4.1 (1.5 - 11.23)
≥ 2	32 (23.7)	103 (76.3)	3.41 (1.36 - 8.62)	3.67 (1.37 – 9.83)
Income				
< 10,000 baht/month	11 (12.6)	76 (76.4)	1	1
≥ 10,000 baht/month	51 (23.9%)	162 (76.2)	2.16 (1.07 - 4.41)	11.67 (0.74 - 3.76)
Contraception use				
No	5 (9.6)	47 (90.4)	1	1
Yes	57 (23)	191 (77)	2.81 (1.07 -7.39)	2.24 (0.82 – 6.18)
Attitude of cervical cancer screening				
Number of parities				
0	46 (61.1)	28 (38.9)	1	1
1	79 (85)	14 (15)	3.59 (1.71 – 7.53)	3.39 (1.55 – 7.42)
≥ 2	103 (76.3)	32 (23.7)	2.04 (1.1 – 3.8)	2.11 (1.07 – 4.19)
History of sexual intercourse				
No	7 (50)	7 (50)	1	1
Yes	219 (76.6)	67 (23.4)	3.27 (1.11 – 9.65)	3.36 (1.07 – 10.67)

**Table 4:** Factors Associated Cervical Cancer Screening Practice

Variable	Practice on cervical cancer screening			Crude Odds ratio^1^ (95%CI)	Adjusted Odds ratio^1^ (95%CI)	Crude Odds ratio^2^ (95%CI)	Adjusted Odds ratio^2^ (95%CI)
	Adequaten (%)	Inadequate n (%)	Never n (%)				
Age							
< 40	41 (61.2)	12 (17.9)	14 (20.9)	1	1	1	1
40-49	105 (76.6)	24 (17.5)	8 (5.8)	4.48 (1.75–11.48)	3.26 (1.02-10.37)	3.5 (1.15-10.63)	3.01 (0.89-10.21)
≥ 50	86 (70.8)	19 (19.8)	9 (9.4)	2.58 (1.03-6.49)	2.35 (0.67-8.32)	2.46 (0.81-7.44)	2.48 (0.64-4.96)
Age of first							
intercourse							
< 20	89 (57.4)	23 (18)	21 (14.2)	1	1	1	1
≥20	129 (84.9)	32 (18.7)	10 (6.6)	1.49 (1.43-7.1)	1.10 (1.01-1.19)	1.48 (0.61-3.59)	0.89 (0.31-.252)
CD4 level							
< 500 cell/mm^3^	85 (57.4)	42 (28.4)	21 (14.2)	1	1	1	1
≥ 500 cell/mm^3^	129 (84.9)	13 (8.6)	10 (6.6)	3.19 (1.43-7.1)	3.41 (1.29-8.99)	0.65 (0.24-1.72)	0.60 (0.21-1.79)
Number of parities							
0	40 (55.6)	17 (23.6)	15 (20.8)	1	1	1	1
1	74 (79.6)	14 (15.1)	5 (5.4)	5.55 (1.87-16.39)	3.33 (0.96-11.49)	2.47 (0.71-8.49)	2.27 (0.61-8.5)
≥ 2	100 (74.1)	24 (17.8)	11 (8.2)	3.41 (1.44-8.06)	2.02 (0.71-5.78)	1.93 (0.71-5.21)	1.56 (0.51-4.79)
Advised about cervical cancer screening							
No	9 (37.5)	6 (25)	9 (37.5)	1	1	1	1
Yes	205 (74.3)	49 (17.8)	22 (8)	9.32 (3.35-25.92)	6.23 (1.84-21.07)	3.34 (1.6-10.54)	2.62 (0.75-9.19)
Attitude regarding cervical cancer screening							
Poor	32 (43.2)	25.3 (33.8)	17 (23)	1	1	1	1
Good	182 (80.5)	30 (13.3)	14 (6.2)	6.91 (3.1-15.38)	5.7 (2.23-14.55)	1.46 (0.6-3.53)	1.35 (0.51-3.59)

Crude Odds ratio^1^, Compared adequate with never screening; Crude Odds ratio^2^, Compared Inadequate with never screening; Adjusted Odds ratio^1^, Compared adequate with never screening; Adjusted Odds ratio^2^, Compared Inadequate with never screening

**Table 5:** The Attitude or Reasons why Women have Inadequate Cervical Cancer Screening

Attitude or reason for ignoring screening	Number (%)
Self-perception (answer more than 1)	
Unnecessary	22(25.58)
No risk	25(29.07)
Lack of symptom	36 (41.86)
Fear of pain	31 (36.04)
Embarrassment	36 (41.86)
Fear of the results	31 (36.05)
Health care-provider (answer more than 1)	
Bad impression with health services	11 (12.79)
Procedure (answer more than 1)	
Expensive cost of screening	24 (27.91)

## Discussion

Only 20.7% of the 300 HIV-infected women in our study had sufficient knowledge about cervical cancer screening. However, 75.3% of participants had good attitude scores and 71.3% practiced adequate cervical screening. These results are similar to those of a study by Olivia et al., (2016). in which only 21.6% of HIV-infected women demonstrated adequate knowledge of cervical cancer screening. By contrast, a study in Kenya found that more than 90% of participants knew about cervical cancer screening (Rosser et al., 2015).

Although most of our participants demonstrated inadequate knowledge, they were aware of the factors that increase the risk of cervical cancer (HIV infection, sexual intercourse, smoking, and parity). However, most (65%) misunderstood that cervical cancer patients have prodrome symptoms before the disease reaches an advanced stage. In addition, only 35% knew that the HPV vaccine was recommended to prevent cervical cancer, which is higher than in Nigeria (3.1%) but lower than in Belgium (50%) (Donders et al., 2008; Rabiu et al., 2011). The factors that were associated with adequate knowledge holding a bachelor’s degree or higher, income > 10,000 baht per month, multiparity, and contraception use, a finding that is consistent with those of a previous study conducted in Laos (Sichanh et al., 2014). 

Seventy-five of participants had high attitude scores, most of whom (83%) indicated that they were satisfied with their routine cervical cancer screening, and 70% of whom strongly desired screening. Four of the major reasons for inadequate screening were embarrassment, lack of symptoms, fear of pain, and fear of the test results. Approximately 80% of HIV-infected women were aware that they were at greater risk of developing cervical cancer, which is higher than in a study conducted Songkla province in southern Thailand (66%) (Charoenmak et al., 2013). These results were similar to those of a study conducted in Laos, which found that awareness of cervical cancer was four times higher in HIV-infected women than in those without HIV (Sichanh et al., 2014).

In a previous study by Rukrungtan et al., (2017) 71% of participants practiced adequate cervical screening according to Thailand’s national guidelines regarding HIV/AIDS treatment and prevention, 18% practiced inadequate screening (low frequency), and 10% had never undergone screening. However, the previously mentioned study in Songkhla found that 83% of participants practiced adequate screening. The reason for this disparity may be that frequency of screening was not included in the previous study’s analysis (Charoenmak et al., 2013). Other studies conducted in Canada, Italy, and the United States found the percentage of HIV-infected women who received adequate screening to be 58%, 61%, and 77%, respectively (Maso et al., 2010; Oster et al. 2009; Pamela et al., 2010). However, studies in Ethiopia and Laos found the rate of adequate screening to be less than 25% (Erku et al., 2017; Nega et al., 2018; Sichanh et al., 2014). These differences may be due to variation in sociocultural, educational, or economic conditions. In addition, non-HIV-infected women in Thailand appear to have lower rates of adequate screening than those with HIV. Studies in northeast Thailand and Bangkok found that only 41% and 42%, respectively, of non-HIV-infected women underwent adequate screening (Chaowawanit et al., 2016; Mongsawaeng et al., 2016).

We found that the women who had children were 1.8 times more likely to be screened for cervical cancer. Similar results were found in Ethiopia, in which women who had children were three times more likely to be screened. This could be due to women with children visiting healthcare facilities more often than those without (Budkaew and Chumworathayi, 2014). In addition, HIV-infected women with CD4 ≥500 cell/mm^3^ were three times more likely to undergo adequate screening than those with CD4 <500 cell/mm^3^. This may be due to higher CD4 count leading to greater adherence to HIV treatment and cervical cancer screening (Suwannanobon et al., 2018). 

Participants who had been advised about cervical cancer screening were nine times more likely to undergo adequate screening compared with those who had not. Women with high attitude scores were nearly seven times more likely to undergo adequate screening. This suggests that all health care providers, especially gynecologists and those in infectious disease clinics, should inform HIV-infected women about their greater risk, reasons to undergo screening, and screening methods. They should also work to clear up any misperceptions regarding cervical cancer screening, such as those involving embarrassment, fear of pain, and fear of test results, and encourage these patients to undergo screening regularly.

*Strengths and limitations*

To our knowledge, this is the first study in Thailand to evaluate the knowledge, attitudes, and practices regarding cervical cancer screening among HIV-infected women. In addition, this was a large trial study that revealed the major reasons that HIV-infected women did not undergo cervical cancer screening. One limitation of this study was that the data may not represent all HIV-infected women in Thailand. Moreover, data were gathered via a self-administered questionnaire and not verified through medical records, which may have resulted in recall bias with regard to screening practice. 

*Implications for practice and further research*

Our results suggest that many HIV-infected women undergo inadequate screening, mainly due to misperceptions regarding cervical cancer. This data may be useful in the development of government policy aimed at educating HIV-infected women with regard to screening in order to reduce the incidence of invasive cervical cancer. 

In conclusion, we found that 71% of participants underwent adequate cervical cancer screening and that the factors that associated with screening were parity, CD4 levels, whether or not patients had been advised about screening, and attitude toward screening. In order to promote cervical cancer screening in HIV-infected women, all healthcare providers should inform their patients about screening, correct any misconceptions, and ensure that screenings are being performed. Routine cervical cancer screening in HIV clinics should be included in a national prevention program in order to ensure that precancerous cervical lesions are detected early and appropriate treatment can be performed.

*Author contributions*

All of the authors participated in the writing of this paper and have read the finished manuscript.

*Declaration of Competing Interest*

The authors declare that they have no known competing financial interests or personal relationships that could have appeared to influence the work reported in this paper. 
